# CD99 tumor associated antigen is a potential target for antibody therapy of T-cell acute lymphoblastic leukemia

**DOI:** 10.37349/etat.2024.00207

**Published:** 2024-02-19

**Authors:** Kamonporn Kotemul, Watchara Kasinrerk, Nuchjira Takheaw

**Affiliations:** Istituto Nazionale Tumori-IRCCS-Fondazione G. Pascale, Italy; ^1^Division of Clinical Immunology, Department of Medical Technology, Faculty of Associated Medical Sciences, Chiang Mai University, Chiang Mai 50200, Thailand; ^2^Biomedical Technology Research Center, National Center for Genetic Engineering and Biotechnology, National Science and Technology Development Agency at the Faculty of Associated Medical Sciences, Chiang Mai University, Chiang Mai 50200, Thailand

**Keywords:** CD99 molecule, CD99 antibody, cancer immunotherapy, T-cell acute lymphoblastic leukemia, antibody drug, T-cell malignancy

## Abstract

Monoclonal antibodies (mAbs) are an effective drug for targeted immunotherapy in several cancer types. However, so far, no antibody has been successfully developed for certain types of cancer, including T-cell acute lymphoblastic leukemia (T-ALL). T-ALL is an aggressive hematologic malignancy. T-ALL patients who are treated with chemotherapeutic drugs frequently relapse and become drug resistant. Therefore, antibody-based therapy is promising for T-ALL treatment. To successfully develop an antibody-based therapy for T-ALL, antibodies that induce death in malignant T cells but not in nonmalignant T cells are required to avoid the induction of secondary T-cell immunodeficiency. In this review, CD99 tumor associated antigen, which is highly expressed on malignant T cells and lowly expressed on nonmalignant T cells, is proposed to be a potential target for antibody therapy of T-ALL. Since certain clones of anti-CD99 mAbs induce apoptosis only in malignant T cells, these anti-CD99 mAbs might be a promising antibody drug for the treatment of T-ALL with high efficiency and low adverse effects. Moreover, over the past 25 years, many clones of anti-CD99 mAbs have been studied for their direct effects on T-ALL. These outcomes are gathered here.

## Introduction

Monoclonal antibodies (mAbs) are biological products. They are now approved as an effective drug for targeted immunotherapy in several cancer types. In 2018, the worldwide market for therapeutic mAbs reached a valuation of US $115.2 billion. It is expected to experience substantial growth and generate revenue of US $300 billion by 2025 [1]. Due to their high specificity, mAbs can specifically recognize the target antigen on cancer cells in variable regions. The mAbs can induce anti-cancer activities through several mechanisms of action. The mAbs are used as magic bullets to interact with their targeted surface molecules expressed on cancer cells. The mAbs that bind to cancer surface molecules (tumor antigens) can induce cancer cell death or inhibit cancer growth. Moreover, the fragment crystallizable (Fc) region of mAbs can enhance immune effector functions to eradicate cancer cells. The engagement of the host immune effectors with the Fc region of mAbs, including complement, natural killer (NK) cells, and monocytes or macrophages, can mediate complement-dependent cytotoxicity (CDC), antibody-dependent cellular cytotoxicity (ADCC), and antibody-dependent cellular phagocytosis (ADCP), respectively [2]. As a result of several action mechanisms of mAbs in cancer eradication, antibody drugs have become a targeted cancer immunotherapy that are more clinically effective than some other chemical drugs. Nowadays, around 100 mAbs have been approved by the Food and Drug Administration (FDA) for the treatment of various solid tumors and hematological malignancies [3]. In addition, there are several therapeutic mAbs that are currently being tested in early- and late-stage clinical trials [4]. However, so far, no antibody has been successfully developed for certain types of cancer, including T-cell acute lymphoblastic leukemia (T-ALL) which is an aggressive form of hematologic malignancies accounting for 15% of pediatric and 25% of adult acute lymphoblastic leukemia (ALL) cases [5–7]. One of the major obstructions in the development of a mAb therapy for T-ALL has been the fact that the molecules targeted by antibodies are mostly tumor associated molecules, which always share their expression between nonmalignant T cells and malignant T cells [8]. The shared expression of surface molecules leads to the killing of nonmalignant and malignant T cells by the antibodies and eventually the induction of secondary T-cell immunodeficiency [8, 9]. T-cell immunodeficiency triggered by antibody treatment may give rise to opportunistic infections and/or the reactivation of latent infections, thereby precipitating life-threatening situations [10]. Consequently, to avoid the induction of immunodeficiency, antibody drugs that kill only leukemic cells without affecting normal cells need to be established. The discovery of potential target molecules is a key factor in the successful development of antibody therapy in T-ALL. In this review, CD99 tumor associated antigen, which is highly expressed on malignant T cells, is proposed as a potential target for antibody therapy of T-ALL. Many clones of anti-CD99 mAbs have been studied over the past 25 years regarding their direct effects on T-ALL.

## CD99 molecule and expression

Human CD99, also known as MIC2 or E2, is encoded by the *MIC2* gene located in the human pseudoautosomal region 1 (PAR1) on the distal short arms of both human X and Y chromosomes (Xp22.33-Xpter and Yp11-Ypter) [11]. Regarding CD99 expression, an erythroid-specific quantitative polymorphism is found to be co-regulated with the Xga blood group polymorphism. The co-expression of the X-linked *MIC2* and *XG* genes appears to be regulated at the transcriptional level by a singular XGR locus situated in the pseudoautosomal region of the sex chromosomes, downstream of the *MIC2* gene [12]. While the Xga antigen shares 48% homology with human CD99, CD99 has not been reported to share homology with other human proteins [13].

**Figure 1:**
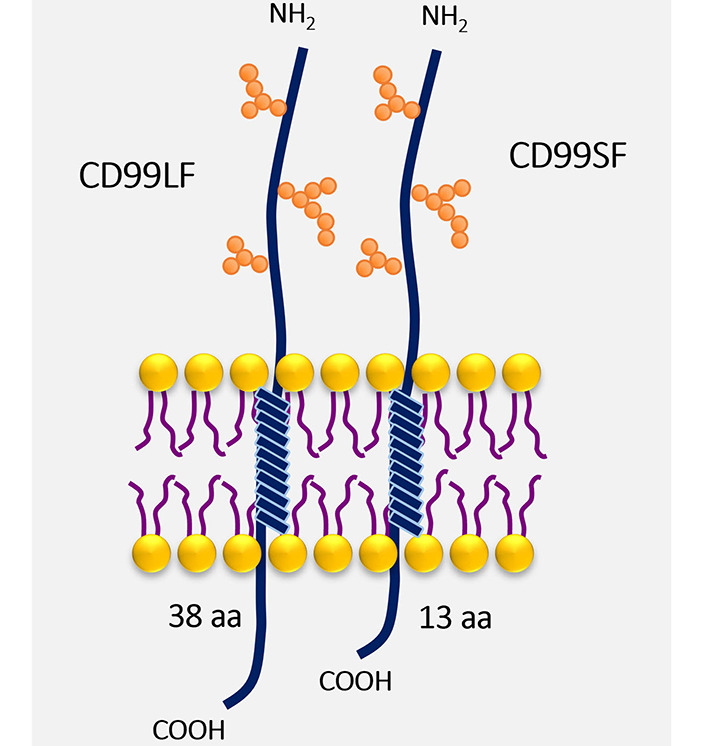
CD99 isoforms. The structure of CD99, a type I transmembrane protein, with O-linked oligosaccharide chains (orange spheres) is shown. The different sizes at the cytoplasmic part of CD99LF and CD99SF are demonstrated. aa: amino acid

CD99 is a type I transmembrane protein, which has highly sialylated glycoprotein with O-linked oligosaccharide chains and absent N-linked oligosaccharide chains [14]. *CD99* gene encodes two isoforms with different sizes at the cytoplasmic part resulting from alternative splicing (Figure 1). The CD99 long form (CD99LF), or wild-type form, contains 185 amino acids and has a molecular weight of 32 kDa. The cytoplasmic part of CD99LF contains 38 amino acids. The alternative splicing CD99 short form (CD99SF), or truncated form, is a 28 kDa glycoprotein that contains 161 amino acids. The cytoplasmic tail of CD99SF consists of 13 amino acids [15]. The alternative splicing CD99SF is the 18-base pair (bp) insertion on the *CD99* gene at the interface of exons 8 and 9 leading to an in-frame stop codon [16]. As a result of the alternative splicing process, the putative phosphorylation sites for serine at amino acid residue 168 and threonine at amino acid residue 181 are absent in CD99SF. The serine 168 is present only in CD99LF, which is an important site for protein kinase C alpha (PKCα) phosphorylation. The PKCα phosphorylation at serine 168 of CD99LF is implicated in the oncosuppressive function of CD99 in prostate cancer and osteosarcoma cells [17].

CD99 exists in two isoforms, which have an intrinsic capability to naturally undergo dimerization on the surfaces of cells and act as a receptor upon stimulation [18]. The CD99 isoforms are expressed in a cell type-specific manner and regulate distinct CD99 functions [13].

The CD99 molecule is broadly expressed on human hematopoietic cells and non-hematopoietic cells, as well as several types of cancer cells [13, 14]. CD99 is expressed at different expression levels depending on cell type. For hematopoietic cells, the CD99 expression levels are likely related to maturation and differentiation stages. High levels of CD99 are expressed on cortical thymocytes while low levels of CD99 are expressed on medullary thymocytes [19, 20]. Furthermore, high levels of CD99 were demonstrated to be expressed on activated/memory T cells and activated B cells [21, 22]. Indeed, each differentiation stage of B cells was exhibited to have different CD99 expression levels. The CD99 expression was the highest in immature pre-B1 stage. During B cell maturation, levels of CD99 expression are reduced from pre-B1 transition to pre-B3 stages. Eventually, low levels of CD99 expression remain on naive B cells [22, 23]. However, upon naive B cell activation and differentiation, CD99 is upregulated in plasma cells that are terminally differentiated B cells [24]. In addition, high levels of CD99 expression were observed in the immature stages of granulocytic lineages. During granulocytic maturation, CD99 expression levels were downregulated leading to CD99 negative/dim on neutrophils in peripheral blood cells [22]. Moreover, monocytes and NK cells also expressed CD99 [19, 22]. For cancer, the alteration of CD99 expression levels has been demonstrated in a broad range of cancers. CD99 plays a role in either oncogenic or oncosuppressive functions depending on the cellular context [13]. CD99 exhibits an oncogenic function, which showed strong expression levels on various cancer cell types, including T cell lineage leukemia/lymphoma, Ewing sarcoma (EWS), breast cancer, acute myeloid leukemia (AML), and myelodysplastic syndromes (MDS) [25–27]. Particularly in T-ALL cells, CD99 was reported to have expression levels at the cell surface about seven times higher than normal T cells, which can serve as a marker for assessing minimal residual disease (MRD) [28]. The mAbs targeting CD99 expressed on these cancers were found to be directly cytotoxic in inducing cancer apoptosis. Furthermore, anti-CD99 mAb was successfully used to inhibit tumor growth in mouse xenografts of EWS, AML, and mantle cell lymphoma [25, 29, 30]. Apart from the direct killing effect, the CD99 antibody possesses the capability to disrupt the interaction between leukemic cells and meningeal cells. The interaction of these cells enhances leukemia chemotherapy resistance. The CD99 antibody that disrupts this interaction can restore sensitivity to chemotherapy in patients who have a relapse in the central nervous system (CNS) [31]. Therefore, CD99 has been proposed to be a potential therapeutic target for an antibody drug in CD99 overexpressed cancers.

## Functions of CD99 in T cell regulation

CD99 is a multifunctional molecule involved in various biological processes, including cell differentiation, cell adhesion, trans-endothelial migration of leukocytes, T cell regulation and protein trafficking, cell apoptosis, and tumorigenesis [13]. In this section, the roles of CD99 in T cell regulation are highlighted. The differential expression of CD99 isoforms is correlated with different stages of T cell maturation [15]. The co-expression of CD99LF and CD99SF is found in the double positive of CD4 CD8 early stage thymocytes, whereas the single CD99LF expression is found in late-stage thymocytes and peripheral mature T cells. Different expressions of CD99 isoforms produce different effects [15]. The functions of CD99 during thymic selection has been elucidated. CD99 was demonstrated to be a transporter molecule. It was revealed that CD99, upon T cell activation, is associated with various accessory molecules. These accessory molecules are transported and gathered at the immunological synapse, which is a site of T cell and antigen presenting cell contact. Upon CD99 engagement, the T cell receptor (TCR), major histocompatibility complex class I (MHC I) and MHC II molecules are upregulated at the cell surface on the thymocyte, especially at the immunological synapse. The upregulation of these molecules might be implicated in thymic positive selection. The translocation of these accessory molecules stored in the cytoplasm to the cell surface occurs through the induction of actin polymerization [32]. CD99 is required for trafficking MHC I from trans-Golgi to plasma membrane when cells respond to interferon-gamma (IFN-γ) [33]. CD99 interacts with MHC I molecule by hydrophobicity of valines amino acid residue in its transmembrane domain as well as CD99 binding to p230/golgin-245 anchored in trans-Golgi membrane leading to the trafficking of MHC I from trans-Golgi to the cell surface [33]. In CD99-deficient cells, the expression of MHC I at cell surface is downregulated. This is caused by a defect in the transportation of this molecule from the Golgi membrane to the plasma membrane [34]. In addition, the roles of CD99 in T cell activation have been reported. The association of CD99 with MHC I, MHC II, and tetraspanin CD81 molecules is found in the lipid raft [35]. Upon T cell activation, these important accessory molecules are translocated to the immunological synapse [35]. CD99 is proposed to be a costimulatory molecule during T-cell activation and a regulator of cytokine production. The co-ligation of CD99 and CD3 increases intracellular calcium ion (Ca^2+^) and induces the production of T helper 1 (Th1) cytokine, including tumor necrosis factor alpha (TNF-α) and IFN-γ [36]. The downstream signaling of CD99 seems to be different from that of the major costimulatory molecule CD28. The engagement of CD99 costimulatory molecule affects TCR-mediated activation of mitogen-activated protein (MAP) kinase, especially c-Jun N-terminal kinase (JNK) and activator protein-1 (AP-1). Unlike CD28, the engagement of CD28 activates the phosphorylation of lymphocyte-specific protein tyrosine kinase (Lck) [37]. Moreover, the cooperation of CD99 and CD3 induces translocation of the TCR ζ chain into the lipid raft, and this event leads to the enhancement of TCR ζ-mediated signal 1. The distinct signals on T cell activation via CD99 co-stimulation and CD28 co-stimulation have been reported [38]. In contrast, the ligation of CD99 by certain clones of anti-CD99 mAb has inhibitory effects to suppress T cell proliferation, CD25 expression, and Th cytokine production [35, 39]. Targeting CD99 reduces the antigen presentation activity on monocytes, resulting in the suppression of T cell functions [39].

## Direct effects of mAbs against CD99 in T-ALL

T-ALL is an aggressive hematologic malignancy that is characterized by clonal growth of immature T-cell progenitors [40]. T-ALL represents 15% of ALL cases, which accounts for 10–15% of ALL children and 20–25% of adults [5–7]. Current treatment of T-ALL is based on multiagent chemotherapy [7]. Although intensive combinations of chemotherapy with or without local radiotherapy demonstrated a high survival rate of approximately 60–90% after receiving frontline therapy. However, the utilization of multiagent chemotherapy comes with a broad spectrum of adverse effects [41]. Chemotherapy often brings challenging side effects associated with its mechanisms of action. Most chemotherapeutic agents induce DNA damage [42]. This mechanism impacts not only cancer cells but also rapidly dividing normal cells in tissues, e.g., the bone marrow, gastrointestinal tract, and hair follicles [42]. The non-selective cytotoxicity of chemical drugs, targeting both cancer and normal cells, results in a narrow margin of safety and makes side effects more pronounced, including myelosuppression, mucositis, nausea, vomiting, diarrhea, alopecia, and fatigue. Additionally, the induced immunosuppression heightens the risk of infections [43]. Moreover, about 20% of pediatric and 40% of adult patients will relapse and become chemotherapeutic drug resistance [44]. The overall survival rate of relapsed patients remains poor, with approximately 3–12% of relapsed patients [45]. To deal with relapsed and/or refractory cases, the exploration of antibody therapies has been a crucial avenue. Two mAbs specifically targeting CD38, isatuximab and daratumumab, offer promised options for individuals facing relapsed and/or refractory multiple myeloma (MM) [46]. Rituximab, anti-human CD20 antibody, has gained approval for the treatment of relapsed and refractory non-Hodgkin’s lymphoma (NHL), particularly in combination with CHOP chemotherapy (cyclophosphamide, doxorubicin, vincristine, prednisone) [47]. However, so far, the available antibody drugs for T-ALL treatment remains limited. Therefore, there is a need for the development of novel antibodies for T-ALL, especially in the treatment of relapsed/refractory patients and patients with chemotherapy resistance.

As of now, several antibodies are undergoing clinical trials for T-ALL, including both approved antibody drugs and novel antibodies. The FDA-approved anti-CD38 mAbs used for MM, daratumumab, and isatuximab, are currently being investigated in clinical trials for T-ALL patients [48]. Additionally, the anti-CC chemokine receptor 4 (CCR4) antibody KW-0761 is undergoing a phase II clinical trial to assess efficacy, safety, and pharmacokinetic profiles in CCR4-positive adult T-cell leukemia/lymphoma [49]. Furthermore, immune checkpoint inhibitors, such as the anti-programmed cell death-1 (anti-PD-1) mAb nivolumab, are in phase II trial studies for the treatment of patients with human T-cell leukemia virus (HTLV)-associated T-cell leukemia/lymphoma [50]. Consequently, the exploration of candidate antibodies for T-ALL remains a critical aspect of ongoing research.

Currently, targeted molecules for antibodies in the treatment of T-ALL are still limited. Most antibodies target on molecules that are found both malignant T cells and normal cells [51]. The shared expression of surface molecules results in the elimination of both malignant and non-malignant T cells through antibodies, subsequently causing the development of secondary T-cell immunodeficiency [10].

Therefore, the studies on novel targeted CD molecules will be helpful for the development of successful targeted antibody therapy [52]. CD99 tumor associated antigen is proposed to be a new potential target for antibody therapy of T-ALL.

**Figure 2:**
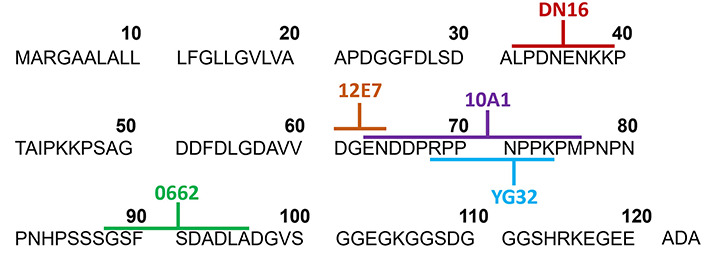
Epitopes recognized by anti-CD99 mAbs. The amino acid sequences of CD99 extracellular domain with a signal sequence are shown. The distinct CD99 epitopes at CD99 extracellular domain recognized by several clones of anti-CD99 mAbs (as indicated) are labeled with different colors. ADA: 3 amino acid of CD99 residues 121–123

In T-ALL, CD99 plays major roles in cell adhesion and cell apoptosis. Interestingly, ligation of CD99 by antibody induces death of immature thymocytes and malignant T cells but does not affect mature cells [53, 54]. The reason is likely that CD99 is extensively expressed in T-ALL but lowly expressed in mature peripheral T cells [27]. Apart from the differential expression levels of CD99 in immature and mature T cells, it is probably dependent on CD99 isoform expression in each stage of cells. The differential expression of two CD99 isoforms is correlated with the stage of T maturation [15]. Both CD99LF and CD99SF can be found on the T cell surface during the immature stage and malignant T cells, whereas single expression of CD99LF is found in the mature stage or peripheral blood cells [15]. The differential expression of CD99 isoforms is involved in the distinct outcomes [15, 17]. The ligation of CD99LF and CD99SF co-expression in CD99 deficient-Jurkat cells (representative of T-ALL) by mAb can induce apoptosis, while ligation on CD99LF induces homotypic aggregation that is not sufficient to induce apoptosis. The proposed mechanism is that both CD99LF and CD99SF form a covalent heterodimer within the glycosphingolipid raft and induce sphingomyelin degradation leading to cell death [15]. Several anti-CD99 mAbs were studied for their direct effects on T-ALL. Anti-CD99 mAb DN16 which binds to CD99 residues 32–39, induced apoptosis in Jurkat T-ALL cell line. The mAb DN16 is mouse immunoglobulin G1 (mIgG1) and can induce Jurkat cell apoptosis in conditions with or without the secondary antibody cross-linker [55, 56]. The mAb 0662, which is mIgG3 and recognizes residues 88–97 of CD99 [55], induced apoptosis in Jurkat T-ALL cell line and immature thymocytes [53]. However, anti-CD99 mAb 12E7 (mIgG1) which reacts to CD99 residues 61–64 [55] or anti-CD99 mAb D44 mIgM fails to induce apoptosis in Jurkat cells and immature thymocytes [53]. Anti-CD99 mAb Ad20 (mIgM) rapidly induces apoptosis in Jurkat and MOLT-4 T-ALL cell lines but has no apoptotic effects in SUP-T1 and normal T cells. The anti-CD99 mAb Ad20 was demonstrated to bind to CD99 at a distant region of 0662 epitope [54]. The engagement of Ad20 epitopes swiftly triggers T cell death through a caspase-independent pathway. Recently, Romero et al. [57] developed the human anti-CD99 antibody clone 10A1, which binds to CD99 residues 63–76, and engineered it into a tetravalent version. The study highlighted the crucial role of antibody valency in ligation of CD99 inducing cytotoxicity. The tetravalent 10A1 exerted cytotoxicity effect against the T-ALL cell line KOPT-K1 but not normal peripheral blood mononuclear cells (PBMCs). Additionally, antibodies with different valencies, including bi-, tri-, and tetravalent antibodies, were generated and compared. Two or more valencies induced homotypic cell aggregation. However, antibodies with a valency of three or more are required for inducing cytotoxicity on T-ALL cells. This finding suggests that at least three CD99 molecules need to be clustered by the antibody to induce cytotoxicity [57]. Although multivalent antibodies are required for inducing the apoptotic pathway of CD99 in T-ALL. However, anti-CD99 mAb D44, which is mIgM and has multivalence, did not induce apoptosis of T-ALL [53], whereas anti-CD99 mAb DN16, which is mIgG1, can induce apoptosis of T-ALL [55]. This indicated that the apoptotic effect of mAbs against CD99 depends on the recognition of the specific CD99 epitopes. Jung et al. [55] demonstrated that each CD99 epitope plays a distinct role in T cell apoptosis. Anti-CD99 mAb YG32 recognizes CD99 residues 68–74 [56]. The mAb YG32 (mIgG), which recognizes different epitopes with anti-CD99 mAb DN16 (mIgG) and has a higher affinity than DN16, fails to induce Jurkat cell apoptosis even in the presence of a second cross-linking Ab [55, 56]. Nevertheless, the ligation of YG32 epitope results in homotypic aggregation of Jurkat T cells [55]. Another clone of anti-CD99 mAb named MT99/3 was also demonstrated to fail to induce Jurkat cell apoptosis, but it could induce homotypic aggregation of Jurkat cells [58]. Khunkaewla et al. [59] reported that anti-CD99 mAb MT99/1 and MT99/2, which are mIgM and bind to distinct epitopes of CD99, show different outcomes. The anti-CD99 mAb MT99/1 can induce homotypic aggregation and apoptosis of Jurkat cells but MT99/2 cannot induce these phenomena [59]. In addition, ligation of CD99 using different mAbs also induces different death signals. CD99 ligation inducing cell apoptosis in double-positive thymocytes and Jurkat cells via Fas-independent pathway has been reported [53]. Meanwhile, triggering on a distinct CD99 epitope has been found to induce apoptosis via Fas-mediated pathway in the Jurkat cells [55]. Moreover, it was revealed that apoptosis events in double-positive thymocytes or immature T cell lines occur through caspase-dependent or caspase-independent pathways in response to antibodies targeting different CD99 epitopes [53, 54]. The direct effects of anti-CD99 mAbs in T-ALL as mentioned above are summarized in Table 1 and epitopes recognized by anti-CD99 mAbs are exhibited in Figure 2.

**Table 1:** The direct effects of anti-CD99 mAbs in T-ALL

**Antibody** **name**	**Isotype**	**Epitope**	**Effect on T-ALL**	**Effect on normal cells**
DN16	mIgG1	LPDNENKK	Induces apoptosis	Not performed
0662	mIgG3	GSFSDADLA	Induces apoptosis	Not performed
12E7	mIgG1	DGEN	No apoptosis	Not performed
D44	mIgM	Not reported	No apoptosis	Not performed
Ad20	mIgM	Not reported	Induces apoptosis	No apoptosis
10A1	hIgG1^※^	ENDDPRPPNPPKPM	Induces apoptosis	No apoptosis
YG32	mIgG	RPPNPPK	No apoptosis Induces homotypic aggregation	Not performed
MT99/3	mIgG2a	Not reported	No apoptosis Induces homotypic aggregation	Not performed
MT99/1	mIgM	Not reported	Induces homotypic aggregation and apoptosis	Not performed
MT99/2	mIgM	Not reported	No homotypic aggregation and no apoptosis	Not performed

^※^ Tetravalent antibody. hIgG1: human IgG1

As mentioned above, the intricate outcomes of CD99 antibodies involve triggering specific epitopes. Certain epitopes can prompt cancer cells to undergo programmed cell death without the need for molecule crosslinking. In the case of some epitopes, the clustering of molecules is necessary and is achieved through the use of secondary antibodies or multivalent antibodies. The quantity of clustered molecules also influences the direct killing effects of CD99 antibodies. Conversely, triggering certain epitopes cannot induce cell death. Therefore, the discovery of epitopes targeted by CD99 antibodies that elicit cytotoxicity to malignant T cells but not to non-malignant T cells is extremely valuable. This discovery holds the potential to use CD99 antibodies for T-ALL treatment with minimal adverse effects.

In cancer treatment, the direct effect of antibodies holds great mechanism. These antibodies induce a specific signaling of targeted molecules that might lead to the death of cancer cells while sparing normal cells, ensuring the safety of the treatment [60]. Additionally, the direct effect of the antibody operates independently immune effector functions which is very useful in the treatment of cancer patients with a weakened immune system [61]. However, the direct effect of some antibodies may not be efficient. Certain cancer cells likely resist the direct killing mechanism and still survive [62]. Therefore, antibodies that exert both direct effect and activate immune effector functions may be a promising drug for cancer treatment with more effectiveness.

Chimeric antigen receptor T (CAR-T) therapy, another non-chemotherapeutic strategy, emerged as a highly successful therapy for the treatment of relapsed/refractory B cell malignancies [63]. CAR-T cells targeting the CD99 molecule have also been engineered and demonstrated the eradication of T-ALL without toxicity to normal blood cells [64]. This finding highlights the potential of CD99 as a promising therapeutic target for T-ALL in the development of various types of targeted therapy approaches. Notably, CAR-T cells against CD99 were generated from normal T cells [64]. They may not reflect the fact in the treatment of T-ALL patients with autologous CAR-T cells. The challenge in CAR-T therapy for T-cell malignancies has been reported. The collection of T cells in T-ALL patients for engineering CAR-T cells could be contaminated with malignant T cells leading to unsatisfactory therapeutic efficacy [65, 66]. This problem restricts the development of a successful CAR-T cell therapy for T cell malignancies. Thus, antibody therapy is still the most promising strategy for the treatment of T-ALL.

## Conclusions

CD99 is a tumor associated antigen that shares expression in both T-ALL and normal cells. Nevertheless, CD99 is strongly expressed in T-ALL cells and low expressed in peripheral blood T cells. Co-expression of CD99LF and CD99SF involved in the activation of apoptotic signaling was found only on T-ALL cells. mAbs that specifically bind to certain CD99 epitopes induce apoptosis in T-ALL cells but not in peripheral blood cells. These findings suggested that certain anti-CD99 mAbs might be a promising antibody drug for the treatment of T-ALL with high efficiency and low adverse effects. Thus, CD99 tumor associated antigen is proposed to be a potential target for antibody therapy of T-ALL. However, for the development of anti-CD99 mAbs for T-ALL treatment, a multivalent structure must be considered in some clones of antibodies. The direct killing effect by inducing apoptosis of antibody drugs has the potential to kill cancer cells alone with no immune mediator functions required. This approach would appear to have the advantage of simplicity in immunocompromised cancer patients who have a weakened immune system. Apart from the direct killing effect, the actions of antibodies in enhancing several host immune effectors for cancer elimination have been demonstrated to improve the clinical outcome of patients in several cancer types. Up to now, the actions of anti-CD99 mAbs mediating ADCC, ADCP, and CDC mechanisms via host immune effectors in T-ALL have not been elucidated. To successfully develop an antibody-based therapy for T-ALL, several clones of anti-CD99 mAbs must be investigated for the anti-cancer effects in both direct killing effect and enhancing host immune effectors *in vitro* and *in vivo*. Importantly, those anti-cancer effects must be studied in malignant T cells and compared with peripheral blood cells.

## Abbreviations

ALL: acute lymphoblastic leukemia
CAR-T: chimeric antigen receptor T
CD99LF: CD99 long form
CD99SF: CD99 short form
mAbs: monoclonal antibodies
MHC I: major histocompatibility complex class I
mIgG1: mouse immunoglobulin G1
T-ALL: T-cell acute lymphoblastic leukemia
TCR: T cell receptor

## Declarations

### Copyright

© The Author(s) 2024.
